# Preserving medical confidentiality in Brazilian criminal investigation cases: a narrative review of the principles of bioethics

**DOI:** 10.1016/j.clinsp.2025.100740

**Published:** 2025-08-19

**Authors:** Henrique Martin Zarpellon, Fabiano Bianchi, Henderson Fürst, Rui Nunes

**Affiliations:** a Department of Social Sciences and Health, Faculty of Medicine, University of Porto, Porto, Portugal; b Universidade Federal Fluminense, Rio de Janeiro, RJ, Brazil; c Hospital Israelita Albert Einstein, São Paulo, SP, Brazil

**Keywords:** Medical confidentiality, Principle of autonomy, Principle of beneficence, Principle of non-maleficence, Principle of justice, Criminal investigation

## Abstract

Medical confidentiality is a fundamental element of the doctor-patient relationship, ensuring a secure environment for sharing sensitive information. This narrative review examines the ethical and legal challenges faced by physicians in Brazil when balancing professional secrecy with the legal duty to report, particularly in the context of criminal investigations. Grounded in the core bioethical principles of autonomy, beneficence, non-maleficence, and justice, the study analyzes Brazilian constitutional and infra-constitutional norms, including the Penal Code, the Code of Criminal Procedure, and the Code of Medical Ethics. The methodology is qualitative and descriptive-analytical, based on thematic and interpretive analysis of legal documents, court decisions, professional guidelines, and ethical frameworks. Comparative perspectives from Portugal, the United States, and international declarations (e.g., WMA Geneva Declaration, UNESCO Bioethics Declaration) are also included to situate Brazil’s legal approach in a global bioethical context. The study highlights the risks of breaching confidentiality without just cause, emphasizing real-life cases involving abortion, HIV disclosure, and unlawful access to patient data. Findings indicate that while breaches may be justified in exceptional situations ‒ such as imminent risk to third parties ‒ confidentiality must remain the standard. The article concludes that ethically informed and legally compliant medical practice requires a careful balance between respecting patient privacy and fulfilling public health and judicial responsibilities. Upholding confidentiality strengthens the therapeutic alliance, protects vulnerable individuals, and ensures alignment with both national law and international bioethical standards.

## Introduction

The relationship between doctor and patient is based on trust and professional secrecy, and the patient must be protected by the state, by legal norms, and by the physician. The reason for this protection is simple: the patient needs a safe environment to share his intimate and private information with his doctor to find a diagnosis and treatment for his illness. Without the guarantee of professional secrecy, there would be a risk that the disease would not be diagnosed and, as a result, treatment would be inadequate or lacking.

França.^1^ reports that: “The silence required of physicians is intended to prevent publicity about certain facts known in the practice or in the face of professional practice, the unnecessary disclosure of which would harm the moral and economic interests of patients. An integral part of medical secrecy is the nature of the disease, the surrounding circumstances, and its prognosis. An individual's right to privacy is, therefore, a gain that enshrines the defense of freedom and the security of intimate relationships, by constitutional principle and by privilege guaranteed in the achievement of citizenship as an integral part of their legal ethical heritage. It is a dimension of freedom that does not admit intrusion”.

Medical confidentiality is the rule in Brazil, and it should be considered by every professional in the category. Several legal norms in Brazil have dealt with the issue of professional secrecy. In this article, the Constitution of the Federative Republic of Brazil (CRFB), the Penal Code, and the Code of Medical Ethics, written by the Federal Council of Medicine (CFM), which is the professional council in charge of regulating the activity of medical professionals in Brazil, should be highlighted.

The Constitution of the Federative Republic of Brazil.^2^ established in its article 5, point X, that intimacy and privacy are inviolable, and the right to compensation for material or moral damage resulting from their violation is guaranteed. The CRFB mentioned intimacy and privacy. This was no accident since intimacy and privacy are different. Marques,^3^ quoting Celso Ribeiro Bastos, teaches that “the right to privacy consists in the ability of each individual to prevent the intrusion of strangers into his private and family life, as well as to prevent them from having access to information about the privacy of each individual, and also to prevent the disclosure of information about this area of the existential manifestation of the human being”. The author goes on to inform that “the right to intimacy is the one that protects us from the knowledge of others, reserves to us our own experience. In this way, intimacy would be a kind of privacy”. In health care, there is information that the patient would like to share with his or her family, school, and co-workers. That is the privacy landscape. Patients have the right to choose who they want to share information about their illness with. On the other hand, if you choose to keep such information only with yourself, this is also a right guaranteed by the CRFB. Here you would be in the realm of intimacy.

The current Code of Medical Ethics.^4^ of the Federal Council of Medicine establishes the rule of medical professional secrecy, maintaining the rule of confidentiality in its article 73. However, it immediately establishes three exceptions: except for just cause, legal obligation, or written consent of the patient. The three exceptions are understood. For example, when the law provides for the breach of confidentiality in notifiable diseases due to infectious diseases, the physician will have a legal obligation to notify the health authority because such conduct is provided for by law. While any situation that is not provided by law, but the breach of confidentiality is justified by its nature, would be a just cause or just reason.

To illustrate just cause or just reason, one could create the hypothetical situation of the patient who receives the news of the HIV diagnosis. Confidentiality is what every physician should strive for in the relationship with his or her patient. The environment of your office or your care must be protected; it must be a safe place for the patient to talk about his disease. After the patient is diagnosed with HIV, he or she tells her doctor that he or she does not want this information shared with her husband or wife because of medical confidentiality. The authors would be at an ethical impasse. However, this situation has already been guided by the Brazilian Federal Council of Medicine, through Opinion n° 27/2018,^5^ which guided the breach of medical confidentiality for legitimate reasons or due to the conflict of two principles of bioethics: the principle of patient autonomy in keeping information confidential and, on the other hand, the principle of non-maleficence in not causing harm to others. In this case, although both principles are legitimate, the principle of nonmaleficence overrides the patient's autonomy and justifies the breach of confidentiality for a sexual partner.

Article 73 of the Code of Medical Ethics.^4^ continues to establish the rules for maintaining medical confidentiality, even when the fact is publicly known or the patient has died, or when he or she testifies as a witness (in which case the physician will appear before the authority and declare his or her disability) and in the investigation of suspected crimes. The physician is prevented from revealing a secret that could expose the patient to criminal prosecution.

What is the justification for maintaining rules that guarantee the confidentiality of patient information, even in the face of unlawful conduct on the part of the patient? Is maintaining confidentiality in the doctor-patient relationship a prerogative that must be defended unconditionally? The purpose of this review is to explore the ethical and legal limits that health professionals face when dealing with the obligation to maintain confidentiality of information that could implicate their patients in criminal proceedings. From this perspective, and considering such considerations, the central question that will guide the investigation arises: To what extent is it possible to reconcile the preservation of medical confidentiality in the context of criminal investigations in Brazil with the principles of autonomy and beneficence of bioethics?

It is a theoretical, qualitative, and descriptive-analytical research. From a methodological standpoint, qualitative research is grounded in an in-depth understanding of social reality, achieved by interpreting the meanings that subjects attribute to their experiences. Unlike the neutrality and rigidity of the positivist approach, this methodology is flexible and open to continually reformulating hypotheses as it interacts with the field. It values the natural environment as a direct source of data and emphasizes meaning as an essential element of analysis rather than measurement. Thus, it is an investigative strategy that favors understanding the complexity of reality and the meanings produced in human interactions while respecting the uniqueness of each studied situation.^6^ While for Lima,^7^ qualitative research considers values and contexts as open paths, but never finished, hence its contribution and relevance in the study of problems located in the field of bioethics, thus meeting the principles of autonomy and beneficence in the maintenance of medical confidentiality even in special situations such as the non-exposure of the patient to criminal proceedings. The study was based on a narrative literature review of normative, doctrinal, and jurisprudential sources on medical confidentiality, bioethics, and criminal liability in Brazil and Portugal.

The inclusion criteria were based on the thematic and legal relevance of the identified sources. The authors included doctrinal works, normative documents, judicial decisions, and technical and professional opinions that specifically addressed medical confidentiality in the context of criminal investigations. The authors also included texts that discussed the bioethical principles of autonomy, beneficence, non-maleficence, and justice. Publications from the last ten years written in Portuguese and English were prioritised, especially those from Brazil and Portugal, given the legal-comparative nature of the study. However, the authors also included older institutional documents, such as opinions from the Federal Council of Medicine dating back to 2000, as these remain relevant and continue to guide the ethical conduct of health professionals. Opinion pieces with no normative or scientific basis, publications without access to the full content, and works that did not directly address the issue of medical confidentiality were excluded.

The analysis was conducted through thematic and interpretative reading to identify categories related to the four principles of bioethics and compliance with legal obligations regarding medical confidentiality. A dialogical approach was also adopted between the fields of law and bioethics, which enabled an interdisciplinary interpretation of the analysed normative and ethical conflicts to be constructed.

## Medical confidentiality in the investigation of suspected crimes

According to the Code of Medical Ethics,^4^ medical confidentiality must be maintained even in the investigation of suspected crimes. However, in addition to the Code of Ethics, other Brazilian norms determine the prevalence of medical confidentiality in this situation. This is the case of Article 154 of the Brazilian Penal Code,^8^ which establishes a crime for anyone who, without just cause, discloses a secret of which he or she is aware by virtue of his or her function, service, office, or profession, and the disclosure of which may cause harm to others. Another norm in harmony with the subject under study is that established in article 207 of the Brazilian Code of Criminal Procedure,^9^ when it states that persons who, by virtue of their function, ministry, office, or profession, are required to keep a secret, are prohibited from testifying, unless they are released by the interested party and wish to give their testimony. Although it is the physician's duty to notify the health authorities of cases of infectious diseases^10^ or other conditions defined in the list of the Brazilian Ministry of Health, known as notifiable diseases, the current legislation expressly prohibits the communication of this information to the police authorities. This legal prohibition aims to protect patient privacy and confidentiality by ensuring that public health is prioritized without compromising individual rights.

It is important to note the existence of article 5, third paragraph of the Code of Criminal Procedure,^9^ which establishes that any member of the public who becomes aware of the existence of an offense in which public action is justified, may report it orally or in writing to the police authority, and the latter, after verifying the information, will order an investigation to be initiated. In this regard, however, the jurist França^1^ emphasizes that the physician who has treated and cared for a patient cannot be included among the other persons who have the obligation to bring the knowledge of the unlawful acts to the competent authority. This is due to the professional practice of the doctor.

In Portugal, medical confidentiality is considered a fundamental pillar of medical practice and is protected by various legal and ethical rules. The Portuguese Constitution.^11^ (Article 26) guarantees the right to privacy, which includes the confidentiality of health information. The Portuguese Medical Association's Code of Ethics.^12^ establishes that medical secrecy is an essential condition of the doctor-patient relationship, covering all facts that have come to the doctor's attention during their profession.

However, the duty of secrecy is not absolute. Regulation 228/2019 of the Portuguese Medical Association^13^ provides for situations in which professional secrecy may be waived, such as with the patient's consent, in cases of compulsory reporting of diseases, or when it is necessary to defend the dignity, honor, and legitimate interests of the doctor, patient, or third parties.

In addition, the General Data Protection Regulation (GDPR),^14^ which is applicable throughout the European Union, establishes strict guidelines on the processing of personal data, including health data. The GDPR permits the processing of such data without the explicit consent of the data subject only in specific circumstances, such as when it is necessary to protect the vital interests of the data subject or another individual.

A notable case occurred in Portugal, where Centro Hospitalar Barreiro Montijo was fined €400,000 for GDPR violations, including unauthorized employees having indiscriminate access to patient data. The National Data Protection Commission (CNPD) identified failures to implement adequate technical and organizational measures to protect patients' personal data, emphasizing the importance of restricted access policies and rigorous control over sensitive information.^15^ This case demonstrates that breaches of confidentiality can cause serious harm to patients and violate fundamental rights such as privacy, intimacy, and dignity, even outside the criminal context. In the context of this study, the same logic applies: disclosing health information without a clear and proportionate legal basis compromises trust in the health system and weakens the doctor-patient relationship. This reinforces the importance of preserving medical confidentiality as an ethical and legal principle, even in the context of criminal investigations.

In Brazil, breaching medical confidentiality without express legal authorization or a legal duty, or without just cause, can constitute a criminal and disciplinary offence, as well as contravening the bioethical principles of autonomy, non-maleficence, beneficence, and justice. Confidentiality must be safeguarded and can only be waived in exceptional circumstances, such as when there is an imminent and real risk to the health of third parties, as set out in health legislation and ethical guidelines issued by Medical Councils.

Medical confidentiality is of the utmost importance, and must be protected even when faced with state interests in criminal repression. This is clearly illustrated by the judgment of the United States Supreme Court in Ferguson v. City of Charleston (532 U.S. 67, 2001).^16^ In that instance, the Court deemed the practice of a public hospital conducting toxicology tests on pregnant women without their prior knowledge and consent to be unconstitutional. The objective of this practice was to subsequently provide the results to the police, thereby enabling criminal proceedings to be initiated against the patients. While those responsible claimed a therapeutic purpose, the Supreme Court ruled that the direct involvement of police authorities in the planning, execution, and use of the tests ruled out any claim of “special need” unrelated to criminal repression. The court ruled that, even if the purpose was beneficial, obtaining evidence for investigative purposes without judicial consent or authorization constitutes an unreasonable search and is therefore prohibited by the Fourth Amendment of the US Constitution.

As demonstrated in the case of Ferguson, which mirrors a situation previously reported in Portugal, the utilization of clinical data for punitive purposes, without the requisite consent or clear legal provision, erodes patient confidence in the health system. This, in turn, has a detrimental effect on access to medical care, particularly among vulnerable populations. Extending the therapeutic function of medicine to utilize it as a tool for criminal prosecution contravenes not only the right to privacy, but also the bioethical principles that govern medical practice. Consequently, foreign jurisprudence offers valuable insights for us to consider the ethical and legal boundaries of cooperation between health professionals and police authorities in Brazil.

The provisions of article 66, section II, of the Law on Criminal Misdemeanors of Brazil^17^ do not contradict the guidelines for maintaining medical confidentiality established in this section, since the rule only imposes the duty to report crimes of public action when such an act does not expose the patient to criminal proceedings. Furthermore, the Federal Council of Medicine issued Resolution n° 1605/2000,^18^ establishing strict guidelines for the protection of medical confidentiality. This reaffirmed that the disclosure of information contained in the patient's medical record or file is only admissible with the express consent of the patient, a formal court order or in legally provided cases of compulsory notification of diseases. Even in cases of unconditional public criminal action, the Resolution instructs the physician to refrain from exposing the patient to criminal proceedings, thus preserving the principles of confidentiality and non-maleficence that support ethical medical practice. Therefore, there is no conflict between the duty to report crimes and the obligation of medical confidentiality, as the patient is not placed at risk of criminal liability.

Technical Note SEJUR n° 016/2011^19^ reaffirms the inseparability of the preservation of medical confidentiality and the fundamental rights to privacy and intimacy. It also clarifies that the direct delivery of medical records to the Public Prosecutor's Office without the patient's consent or without a formal court order constitutes a violation of the doctor's ethical and professional duty, in addition to a potential criminal offense. The technical statement of the CFM consolidates the normative and jurisprudential understanding that medical confidentiality cannot be relativized for investigative convenience. It must be treated as a fundamental right and a guarantee of the very dignity of the human person. The guidance preserves the patient's fundamental rights and protects the medical professional from incurring ethical and criminal violations. It reaffirms the balance between the duty of confidentiality and the public interest in elucidating the facts.

## Principle of patient autonomy and medical confidentiality

The principle of autonomy^20^ promotes respect for the ability and right of individuals to make free and informed decisions about their lives and health. In the context of Brazilian law, this principle is particularly respected in relation to norms that protect the confidentiality of patient information, even in the face of criminal investigations, as illustrated by Articles 73 of the Code of Medical Ethics,^4^ 154 of the Brazilian Penal Code^8^ and 207 of the Brazilian Code of Criminal Procedure.^9^

Brazilian law protects patients' autonomy by ensuring that their personal information remains confidential, even in situations that could potentially expose them to criminal prosecution. This allows patients to feel comfortable sharing sensitive information with their physicians, facilitating a relationship of trust that is essential for accurate diagnosis and effective treatment. The existence of these laws reinforces the importance of the doctor-patient relationship as a safe space, encouraging patients to seek help without fear of legal reprisal. This is particularly important in public health issues, such as the treatment of notifiable diseases, where early detection and treatment are critical to preventing the spread of infectious diseases. Although these conditions must be reported to public health authorities, the legislation ensures that such reporting preserves the anonymity of the patient while protecting his or her autonomy and privacy.

Medical confidentiality is essential to ensure that patients feel safe sharing personal information with their physicians. Revealing information that could expose the patient to criminal prosecution without their consent would violate their autonomy and freedom to make decisions about their own health. For example, a patient with a history of substance abuse may be afraid to seek treatment if they learn that their medical information could be used against them in a criminal case, thereby jeopardizing their access to needed health care.

The tension between the protection of professional secrecy in medicine and the legal obligation to report certain unlawful acts illustrates a complex ethical and legal challenge, especially when the authors consider the principle of autonomy in bioethics. This principle, which emphasizes the right of individuals to make free and informed decisions about their health and bodies, is in a delicate balance with Brazilian regulations on the confidentiality of medical information.

The decision of the Sixth Panel of the Superior Court of Justice, which judged a case in which the doctor violated professional secrecy by revealing the criminal practice of a patient who committed the crime of abortion.^21^

The constitutional role of the Superior Court of Justice is to standardize the interpretation of federal legislation throughout the country.^22^ While the decisions made are not legally binding, they do carry significant weight and serve as a point of reference to ensure consistency, stability, and legal certainty in the application of the law. Therefore, the way the STJ addresses cases of medical confidentiality in the context of illicit practices, such as abortion, reflects the prevailing jurisprudential understanding in the country.

In doing so, the doctor not only violated Article 207 of the Code of Criminal Procedure, which prohibits the disclosure of professional secrets obtained in the practice of medicine, but also violated the principle of autonomy by failing to respect the confidentiality of the patient's information. This action, although motivated by suspicion of wrongdoing, ignores the complexity of the doctor-patient relationship and the patient's right to control information about his or her own body and health. The cited case illustrates the importance of considering patient autonomy, a central pillar of bioethics, in the face of legal requirements. When deciding whether to disclose sensitive information, health care providers should consider not only the legal consequences of their actions, but also the ethical impact of those decisions on the lives and well-being of their patients. Brazilian legislation seeks to balance these interests by protecting medical confidentiality to ensure that the trust between doctor and patient is not compromised. This balance is particularly challenging in abortion cases, where issues of bodily autonomy and reproductive rights are at stake. The STJ's decision reaffirms the importance of protecting patient privacy and autonomy, even in the face of actions that may be considered illegal under current law. Thus, the interaction between bioethics and the laws governing medical confidentiality in Brazil reveals a harmonious field in which the protection of patient autonomy must be carefully balanced with the legal obligations of health professionals. This case serves as a critical reminder of the need for clear guidelines and ethical training for physicians to ensure that they can navigate these complex issues while respecting both bioethical principles and legal requirements.

It is important to note that the list prepared by the Ministry of Health lists some diseases as notifiable and determines that the physician has the duty to break confidentiality and inform the health authorities about cases of domestic violence, sexual violence, attempted suicide, and other violence.^23^ This rule does not specify whether these are cases of patients who would commit the crime or whether these patients would be the victims. In any case, the physician is obliged to inform the health authority, but cannot inform the police authorities. Therefore, it is not a rule that contradicts existing legislation.

The principles supporting medical confidentiality in Brazil are aligned with widely recognized international ethical guidelines. However, these guidelines do not directly address maintaining confidentiality in specific situations, such as protecting patients from exposure to criminal proceedings. The updated version of the Geneva Declaration of the World Medical Association^24^ affirms the physician’s commitment to “respecting the secrets entrusted to me, even after the patient’s death”, thereby reinforcing the permanent and unconditional ethical duty of confidentiality. Similarly, the UNESCO Universal Guidelines on Bioethics and Human Rights^25^ emphasize the protection of privacy and the requirement of informed consent in Article 9, linking confidentiality to respect for human dignity and the autonomy of the patient. While these documents do not explicitly address confidentiality in relation to criminal investigations, their principles reinforce the notion that confidentiality is an integral part of the doctor-patient relationship, and that any deviation must be explicitly justified by specific legal standards. Therefore, by upholding professional secrecy, even when there are potential legal implications for the patient, as discussed in this study, the Brazilian legal and deontological system aligns with internationally enshrined bioethical values.

## Principle of beneficence and medical confidentiality

The interaction between medical ethics and Brazilian legislation, particularly about professional secrecy in the context of not exposing patients to criminal proceedings, reveals profound implications for the principle of beneficence in medical practice. This principle,^20^ which emphasizes actions aimed at promoting well-being and preventing harm to patients, is in a delicate balance with legal obligations to keep patient information confidential, even in the face of suspected crimes. Considering the previously mentioned case of a physician treating a patient who is engaged in illegal activities, such as the use of prohibited substances, the principle of beneficence directs the physician to treat the patient with the goal of promoting his or her health and well-being, without moral judgment. The disclosure of this information, based on Article 154 of the Brazilian Penal Code, could have legal consequences for the patient, which contradicts the principle of beneficence by causing direct harm to the patient's well-being. In this context, of the patient's non-exposure to criminal proceedings, it is only up to the physician to take care of and treat his patient. Physicians must adopt a non-judgmental stance and preserve ethical neutrality in the therapeutic relationship, ensuring that the patient's moral conduct does not affect the quality of care provided.

The principle of beneficence is particularly relevant in cases involving complex decisions about maintaining professional secrecy. For instance, in October 2023, the Superior Court of Justice^26^ granted a habeas corpus to dismiss criminal proceedings against a woman accused of attempting an abortion, after the doctor who had treated her at a public hospital had directly called the police, resulting in her arrest. The court emphasized that evidence obtained through the breach of professional secrecy was unlawful, as such medical conduct directly contradicts the ethical and legal principles underpinning the essential doctor-patient relationship of trust. This case demonstrates that the principle of beneficence should extend beyond the immediate clinical setting to protect patients from secondary harm, including unfair legal consequences arising from the improper breach of professional secrecy.

França^1^ asks, “Should the doctor report the patient who has had an abortion to the police authorities?” The renowned jurist answers in the affirmative: “When an abortion is provoked by the patient herself, of her own free will, without pressure, coercion, or insinuation, and she needs medical assistance, the case becomes more urgent because the question of confidentiality becomes only in favor of the patient and not of an agent provocateur. If the secret belongs to the patient, if the maintenance of confidentiality is morally and ontologically in his favor, the breach of confidentiality would be permissible only for his benefit”.

The issue of induced abortion places the physician in a delicate position, requiring an approach that respects the patient's autonomy while promoting her overall well-being. When a patient chooses to have an abortion of her own free will, without pressure or coercion, and subsequently requires medical assistance, the situation requires heightened attention to the principle of beneficence. In this context, the physician's duty is not only to treat the physical consequences of the act, but also to provide emotional and psychological support, while respecting professional confidentiality and protecting the patient's privacy.

Considering the above, the physician should always act with the intention of promoting the best interest of the patient, guided by the principle of beneficence. This implies a medical practice that not only alleviates physical suffering, but also supports the patient emotionally, while respecting his or her choices and privacy. Beneficence therefore, goes beyond the mere application of medical treatments; it encompasses a holistic approach to health, considering the physical, emotional, and social dimensions of the human being.

## Principles of nonmaleficence and fairness in maintaining medical confidentiality

The principle of nonmaleficence in bioethics guides medical practice and the relationship between doctors and patients, as well as their interaction with society and the law.^20^ Article 73 of the Brazilian Code of Medical Ethics,^4^ by establishing that medical confidentiality must be maintained even in the face of investigation of suspected crimes, directly reflects the principle of nonmaleficence, which is based on the premise of not causing harm to others. This ethical imperative ensures that confidential information provided by the patient to the physician in a context of trust and vulnerability is not used in a way that could cause the patient moral, physical, or psychological harm.

In turn, Article 154 of the Brazilian Penal Code^8^ complements this view by penalizing the improper disclosure of professional secrets, reaffirming the importance of confidentiality in professional relationships, including in medicine. The law recognizes the potential harm that such disclosure may cause, in keeping with the principle of non-maleficence, which seeks to avoid harm to the moral and economic interests of patients.

The principle of justice, which seeks an equitable distribution of benefits and risks, is also reflected in these provisions. By prohibiting the disclosure of confidential information to the judicial system without just cause or the express consent of the patient, the legislation seeks to balance the rights of the individual with the needs of the community. This is evident in the treatment of notifiable diseases, where the law allows ‒ and requires ‒ the reporting of certain conditions to health authorities, but not to law enforcement. This distinction ensures that public health is prioritized and that the collective good is promoted without compromising patient privacy and individual rights, reflecting a fair balance between public and private interests. In addition, Article 207 of the Brazilian Code of Criminal Procedure^9^ reaffirms the commitment of the judiciary to protect the confidentiality of sensitive information by establishing exceptions to the obligation to testify in cases where the function, ministry, office, or profession requires the maintenance of secrecy. This not only protects individuals from potential harm resulting from the disclosure of their personal information, but also preserves the integrity of the doctor-patient relationship, which is essential for effective diagnosis and treatment.

The ethical dilemma faced by health care professionals when treating patients who confess to illegal acts, such as attempted murder, highlights the bioethical principles of nonmaleficence and justice, especially in the context of not exposing the patient to criminal prosecution. Consider a scenario in which a physician sees a patient with a severely burned arm. During the consultation, the patient confesses that the burns were the result of an attempt to murder her husband by setting fire to the house while he slept. The patient managed to escape, but not before sustaining severe injuries. This complex scenario presents the physician with the challenge of balancing the obligation to do no harm, to respect the patient's autonomy, and to administer justice in an equitable manner, considering both individual needs and collective rights.

Another emblematic example involving the principle of non-maleficence occurred in Minas Gerais,^27^ where the Court of Justice recognized the legitimacy of breaking medical confidentiality for a just cause. This was in a case involving an HIV-positive patient who had refused to inform his sexual partner about his condition. Based on opinion n° 06/17 of the Regional Council of Medicine of the State of Bahia, the doctor reported the situation to protect the health and life of the potentially harmed third party. This case highlights the importance of the principle of non-maleficence, showing that while professional medical confidentiality is important, exceptional circumstances can justify breaking it to prevent imminent harm to others, balancing individual and collective interests in medical practice and professional ethics.

The principle of non-maleficence is an important factor in medical decisions about disclosing confidential information in criminal contexts. The decision-making matrix is structured around assessing whether disclosing information would cause significant harm to the patient or third parties. From this perspective, the principle of non-maleficence is invoked to avoid both direct harm to the patient, such as unfair exposure to legal penalties, and indirect harm to public trust in the medical profession and the health system in general. Therefore, physicians must carefully consider the potential harm their actions may cause and protect patients and third parties from unnecessary or disproportionate adverse consequences.

Nonmaleficence, which prioritizes the imperative to do no harm, is tested considering the patient's confession. However, knowledge of the criminal act places the physician in a situation where failure to inform the authorities can be seen as a breach of the ethical and social duty to prevent future harm to third parties, configuring an ethical conflict between nonmaleficence and social responsibility. On the other hand, the principle of justice, understood as the search for equal treatment and equitable distribution of health care according to individual needs, suggests that patients deserve to receive adequate medical care regardless of their actions outside the health care environment. Justice, complemented by equity, demands that everyone be given what is due, recognizing the patient's unique needs-in this case, treatment for his burns-without discriminating based on acts committed, without judging the value of the medical professional.

The inclusion of the concept of equity in the principle of justice broadens the discussion by introducing the need to consider the consequences of medical action not only for the patient but for society.

A relevant application of the principle of justice relating to medical confidentiality occurred during the 2020–21 Brazilian wave of the SARS-CoV-2 pandemic.^28^ Legislation was introduced that made it compulsory to notify health authorities of confirmed cases of the disease, enabling the government to implement effective public policies for epidemiological control. This legal obligation represented an explicit exception to individual medical confidentiality and aimed to guarantee distributive justice by adopting measures that benefited the entire community, such as social isolation and equitably organizing the vaccination queue. The prioritization of the most vulnerable groups in the vaccination campaign against the novel coronavirus, including the elderly, obese individuals, and healthcare professionals, was based on the criteria of equity, protection, and greater vulnerability to risk. This strategy reflected the principle of justice, ensuring limited resources were distributed to those who needed them most first, thereby protecting the most vulnerable individuals and mitigating the global impact of the pandemic. This example highlights that exceptional breaches of medical confidentiality can sometimes be morally justified and legally required to protect overriding collective rights, thereby reinforcing the vital role of justice in medical practice and public health bioethics.

The principle of justice informs medical decisions regarding the maintenance or disclosure of confidentiality in criminal contexts, requiring a balanced assessment of individual and collective interests. When applying this principle, health professionals must analyze whether the strict preservation of confidentiality could cause disproportionate harm to society, for example by enabling unlawful acts to continue or failing to protect victims adequately. An example of this legal and ethical requirement can be seen in the Statute of Children and Adolescents (Law n° 8069/1990, Article 13),^29^ which obliges doctors and other healthcare professionals to notify the relevant authorities of suspected or confirmed cases of child abuse. This legal obligation reflects the principle of justice, ensuring special protection for vulnerable individuals and preventing medical confidentiality from being used to conceal or perpetuate abuse against children. It thus ensures a fair and balanced distribution of social protection.

Conscientious objection appears as a possible way out for the health professional who, given the seriousness of the confessed act, feels morally unable to maintain the confidentiality of the confession, if it is not an urgent or emergency case. However, this right of the professional to refuse to participate in procedures that contradict his or her ethical or moral values must be carefully balanced with the patient's right to treatment and confidentiality.

Faced with this scenario, the physician finds himself in a complex decision-making process in which he must prioritize the intrinsic value of the person, seek to do good while avoiding evil, respect the patient's choices, and finally act with justice and equity. This case illustrates the complex interrelationship between the principles of bioethics in clinical practice and highlights the importance of a reflective and contextualized ethical approach that considers all the dimensions involved: personal, professional, and social, to arrive at a fair and just decision. Therefore, the intersection of these norms with the principles of nonmaleficence and justice in bioethics illustrates a conscious effort by the legal and ethical system to protect patients by ensuring that their rights to privacy and confidentiality are respected. This underscores the importance of medical care that not only refrains from causing harm but also promotes fairness and justice by ensuring that all patients, regardless of circumstances, receive dignified and respectful treatment.

## Medical experts and the exposure of secrecy in criminal proceedings

So far, there has been discussion about the doctor-patient relationship, about confidentiality, and about maintaining medical confidentiality even in the circumstances of not exposing the patient to criminal proceedings. However, there is a question about the role of the medical expert who acts on the order of the judge in a criminal case and who decides a criminal case. Do the above-mentioned laws and principles of bioethics, autonomy, and beneficence also apply in this case, protecting the patient and preventing, for example, the medical expert from having access to the medical record?

The Federal Council of Medicine issued a technical advisory to the Brazilian Medical Society that, yes, medical professionals should describe the truth and all the circumstances of what they find, discover, and observe.^30^ It is important to distinguish the existence of two types of medical professionals: one who has established a relationship of trust with the patient, the attending physician, who is therefore prevented from violating professional secrecy and exposing the patient to criminal proceedings, and the other professional, the medical expert, who has no relationship with the patient. The attending physician did not report the patient. He protected the patient's privacy and kept the service confidential. On the other hand, there were other ways to investigate or prosecute and, consequently, to appoint a medical expert. Would this, in turn, be responsible for describing to the judge what was observed in the expert assessment under Article 154?^8^

Although the medical expert does not establish a therapeutic relationship with the person being examined, their status as a healthcare professional means they are subject to the ethical principles of medicine, particularly when dealing with sensitive information that could implicate the patient in criminal proceedings. This creates a significant ethical dilemma: to what extent does the duty to cooperate with the judiciary, acting as a technical assistant to the courts, take precedence over bioethical principles such as non-maleficence, beneficence, autonomy, and justice?

Opinion n° 22/2000 of the Federal Council of Medicine^31^ expressly recognises that providing medical records without the patient's express authorization constitutes illegal coercion and an ethical violation, even in the face of a judicial request. However, an exception is permitted in cases involving unconditional public action, provided the records are made available to the official expert under expert secrecy and there is no risk of implicating the patient in criminal proceedings. Thus, although the expert physician must report the expert findings with technical accuracy, he or she must do so with ethical awareness of the possible harmful repercussions for the person being examined. This dilemma requires a critical and prudent stance that recognizes the limit between cooperation with justice and respect for human dignity. Therefore, the expert's actions, even if legally supported, are not ethically exempt from the moral impacts of their actions, especially when they involve sensitive data, privacy, and possible criminal repercussions. It is up to the expert to carefully weigh the duty to inform and the commitment to fundamental rights, preserving the ethical limits of medicine, including in the forensic sphere.

## Conclusion

Ensuring the confidentiality of medical information in criminal investigations is one of the most significant ethical and legal challenges in contemporary medical practice. This study demonstrated that, even in the face of suspicions of criminal offences, maintaining the confidentiality of patient information is supported by Brazilian constitutional, infra-constitutional, and deontological norms, and is coherently aligned with the bioethical principles of autonomy, beneficence, non-maleficence, and justice. The normative analysis revealed that the Constitution of the Federative Republic of Brazil, the Penal Code, the Code of Criminal Procedure, and the Code of Medical Ethics together form a robust system of protection that limits breaches of medical confidentiality to situations that are legally foreseen, such as compulsory notification to the health authority. At the same time, institutional documents such as the Federal Council of Medicine's opinions and resolutions reinforce the understanding that confidentiality cannot be relativized for investigative convenience and must be treated as a fundamental patient right, even after death or when the patient has been involved in unlawful acts.

In turn, the bioethical approach allowed us to grasp the depth and complexity of the doctor-patient relationship. The principles of autonomy and beneficence justify preserving confidentiality as a means of respecting patients' freedom and protecting their dignity. The principle of non-maleficence acts as a guiding criterion in refusing to expose the patient to criminal proceedings and the subsequent moral, social, and legal consequences. The principle of justice reinforces the need for equitable treatment and respect for individual rights, even when faced with social demands for criminal repression.

Case studies from Brazil, Portugal, and the United States have highlighted the real and tangible risks associated with the improper disclosure of health data or the utilization of such information in criminal proceedings without the necessary legal basis. Case law from the Superior Tribunal of Justice and the firm position of the Federal Medical Council have reaffirmed that any violation of medical confidentiality constitutes an ethical and criminal infraction in the absence of just cause, legal duty, or express consent of the patient. The role of the medical expert has also been emphasized; their technical function does not exempt them from respecting the fundamental rights of the person being examined. Even if they do not have a therapeutic relationship with the patient, they must observe ethical limits, especially when the disclosure of data may lead to the person being examined being held criminally liable.

In short, the confidentiality of medical information must be the norm, with any exceptions being rigorously justified and limited. In light of the challenges posed by the intersection between criminal law and bioethics, medical professionals must have technical knowledge, solid ethical training and legal sensitivity to enable them to make responsible, fair and humane decisions. It is within this framework that truly ethical medicine is sustained: a person-centred approach that promotes health and protects fundamental rights.

## CRediT authorship contribution statement

**Henrique Martin Zarpellon:** Writing – original draft, Methodology, Investigation, Conceptualization. **Fabiano Bianchi:** Writing – review & editing, Supervision. **Henderson Fürst:** Writing – review & editing, Supervision. **Rui Nunes:** Writing – review & editing, Supervision, Methodology, Conceptualization.

## Conflicts of interest

The authors declare no conflicts of interest.
